# A New Approach to Compute the Porosity and Surface Roughness of Porous Coated Capillary-Assisted Low Pressure Evaporators

**DOI:** 10.1038/s41598-018-30090-9

**Published:** 2018-08-03

**Authors:** Poovanna Cheppudira Thimmaiah, Asish Kumar Panda, Upendra Kumar Pandey, Claire McCague, Pradip Dutta, Majid Bahrami

**Affiliations:** 1 Laboratory for Alternative Energy Conversion (LAEC), School of Mechatronic Systems Engineering, Simon Fraser University, Simon, BC V3T 0A3 Canada; 2 Materials Research Centre (MRC), Indian Institute of Science (IISc), Bangalore, 560012 India; 3 Interdisciplinary Centre for Energy Research (ICER), Indian Institute of Science (IISc), Bangalore, 560012 India

## Abstract

The fundamental characteristics of metal coatings that influence heat transfer are porosity and surface roughness. It is a challenge to analyze the porosity and surface roughness due to the inadequate amount of copper per coated area. In this study, a new approach to non-invasively determine the porosity of metal films utilizing a helium pycnometer and computed micro-tomography (CMT) is presented. Furthermore, a telescope-goniometer is used to measure the surface roughness. Experiments are conducted on four varieties of thin film samples coated with copper powder using wire flame and plasma thermal spray coating methods. The porosities of the thin films were determined to be between 39 and 43%. The thermal spray coating increased the hydrophobicity of the surface and the plasma coating created super-hydrophobic surfaces. The new approach establishes that the porosity of thin films can be non-invasively determined and may also be applied to a wide variety of coated surfaces.

## Introduction

In a low-pressure (LP) evaporator, the operating pressure of the refrigerant (water) is low (~1 kPa), and the cooling power generation in a flooded evaporator is negatively affected by the saturation pressure difference along the height of the water column^1,2^. At evaporation temperatures between 5 to 10 °C, nucleate boiling does not occur as it requires a high wall superheat of up to 20 K^2–4^. At low pressure, natural convection determines the heat transfer, which results in low heat transfer coefficients. As a result, large surface areas are required to achieve high thermal conductance. To achieve high heat transfer coefficients, several researchers have studied thin-film evaporation, such as capillary-assisted evaporation, including applying porous metal coatings to fins to increase capillary action and enhance heat transfer^5–8^. While the narrow channels between high-density fins provide necessary wicking to wet the outside surface of the tube, the thin film porous coating leads to double wicking of the water into the pores, leading to high external heat transfer coefficient, *h*_o_.

The fundamental characteristics of metal coatings that influence the heat transfer are: (i) surface roughness, and (ii) the porosity, particularly the open pore porosity. It is a significant challenge to nondestructively analyze open porosity, closed porosity and surface roughness due to the inadequate amount of copper per coated area. The porosity (*P*) is defined as the ratio of pore volume (*V*_pore_) to the total volume (*V*_total_).

1 $$P=\frac{{V}_{pore}}{{V}_{total}}$$

Gas adsorption measurements are often used to quantify open porosity^9,10^. However, for thin films adhered to the surface of a substrate such as copper, the small amount of coated mass relative to the substrate mass creates experimental challenges and leads to high uncertainties. Besides, gas adsorption experiments need grinding of the material to access the closed pores and quantify the total porosity. However, grinding the thin film material is not advisable because of the small amount of material per coated area.

Direct imaging methods can be used to analyze the porosity of thin films, including scanning electron microscopy (SEM) with energy dispersive X-ray spectroscopy (EDX), wavelength dispersive X-ray spectroscopy^11^, focused ion beam SEM (FIB-SEM) and transmission electron microscopy (TEM). Imaging methods allow for quantification of the porosity via visualization and reconstruction of the pore geometry from 2-D projections of thin films cross sections^12^. However, elaborate sample modification is required, which can result in morphological changes to pore. Therefore, a direct imaging process that can visualize non-invasively and nondestructively is desirable.

Mercury intrusion porosimetry (MIP) is another method for characterizing open pores and interconnected pores^13^. However, mercury can react with copper in a process called amalgamation^14^. Therefore, methods involving inert fluids should be considered.

Gas expansion methods that employ Boyle’s law, most notably helium pycnometry, are among the most accurate techniques for measuring porosity^13,15^. An inert gas, rather than a liquid, is used because it penetrates even the finest pores and minimizes the influence of surface chemistry^16^. Helium has a high diffusivity, and therefore affords a useful means for determining the porosity of thin films. However, the pycnometer can only measure the volume of pores accessible to the helium, therefore it can only measure the open porosity of a sample. Therefore, pycnometry has to be combined with another non-invasive method for complete analysis of both open and closed pores.

X-ray computed micro-tomography (CMT)^17^ has become a valued tool for non-destructive 3D visualization and characterization of porous material^18,19^. However, CMT is a relatively expensive technique due to the complexity of the instrument and the required image processing software.

There are several methods to measure the roughness of the coated surface. Atomic force microscopy (AFM) is commonly used for surface roughness measurements, although the measurement area for AFM is usually limited to few micrometers. Stylus based profilometres are also used to measure the surface roughness. Usually for thermally coated surfaces, the roughness is usually a few micrometers and stylus profilometres cannot provide accurate roughness measurement. The stylus is usually few microns in diameter which makes it impossible to penetrate into smaller structures.

The Wenzel method^20–22^ is a simpler, alternative approach, in which the contact angle of the liquid droplet measured on the coated film surface is used to establish the surface roughness. The scale condition of the Wenzel model should be satisfied, that is, the diameter of the droplet (~5 mm) should be three orders of magnitude larger than the surface pores^23^.

In this paper, a new approach to determine the film porosity by utilizing both helium pycnometer and computed micro-tomography (CMT) is presented. The new approach is based on measuring the open pore volume (pycnometer), mass deposition of the film and the thickness of coated films. The mass of the film is converted into film density by dividing by the measured volume of the film (thickness × area). Ultimately, the film porosity is calculated from the measured porous film density and the density of bulk, non-porous material. By using this approach, the open, closed and total porosity of the copper thin films can be distinctly measured without grinding the sample or changing the morphology of pores. To our knowledge, the quantification of porosity of thin films employing pycnometer and CMT has never been reported in the literature. Therefore, vital contributions of this work are the quantification of porosity of copper thin films using pycnometer and CMT. To determine surface roughness, contact angles are measured using a telescope-goniometer and the Wenzel method is used for analysis.

## Methodology

**Figure 1:**
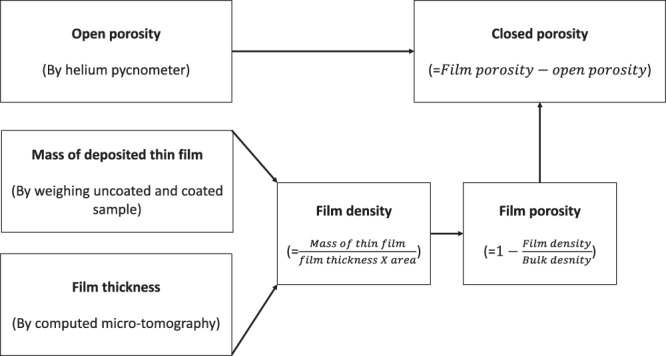
The proposed approach to determine porosity and surface roughness.

The new approach to determine the thin film porosity and surface roughness is illustrated in Fig. 1. The procedure to quantify the porosity is demonstrated on thin-film of copper coated on to a copper substrate. The copper powder was coated using state-of-the-art thermal spray coating techniques^24^, which provide uniform coating thickness. The overall method involves the following steps: the quantification of open porosity by helium pycnometry, the determination of mass deposition by weighing the uncoated and coated samples, and the determination of film thickness non-invasively by CMT. The average density of the film is calculated from the mass deposition of the coated film and measured film thickness. The film porosity is calculated according to Eq. 1 from the measured density of the uncoated sample and the calculated film density. The contact angles on an uncoated and coated copper substrate are measured with a telescope-goniometer and the surface roughness is measured by applying the Wenzel model.

## Experimental Study

### Sample Preparation

Experiments were conducted on five different of samples. Two thermal spray techniques were employed, namely wire flame and plasma spray technology^24^. Two samples were prepared by each technique. Samples from wire flame coating using a flame spray wire gun (Metco 12E) are named W1 and W2 and samples prepared from plasma spray coating using a plasma thermal spray gun (Metco) are named P1 and P2. One uncoated sample was also prepared which serves as reference material to determine the bulk density.

**Figure 2:**
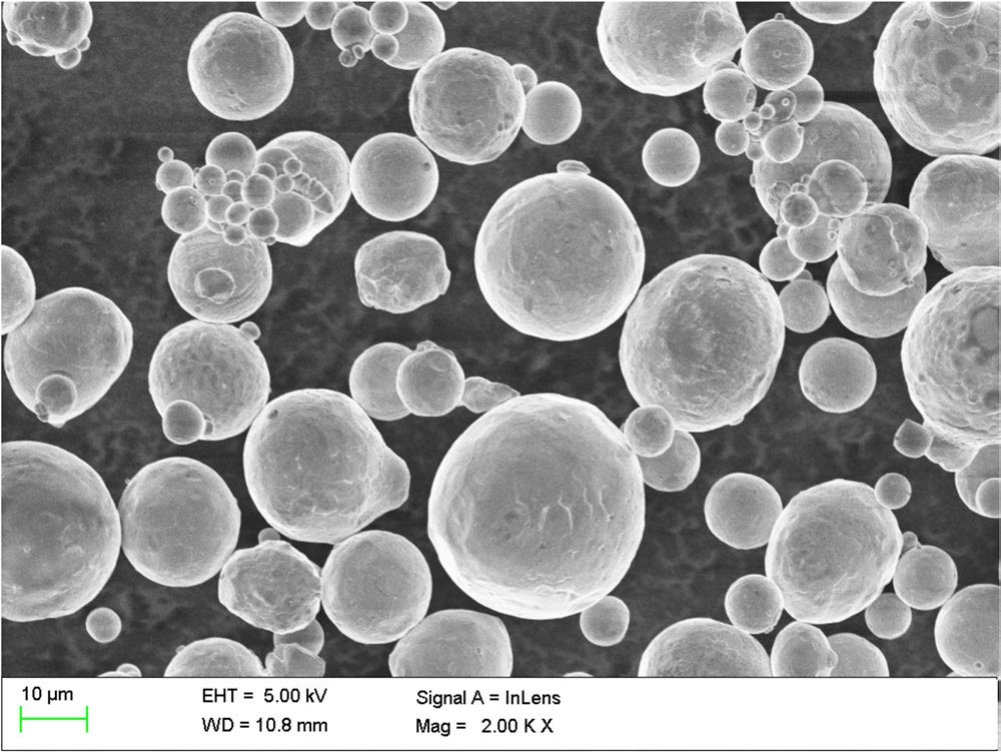
SEM image of copper powder used in the thermal spray method.

Copper (Cu) powder was thermally sprayed onto a 99.9% pure copper sheet of 20 mm × 20 mm and a thickness of 0.7 mm. The SEM image of Cu powder in Fig. 2, shows the range of particle sizes present in the powder. The standoff distance between the sample and the gun was 127 mm. The process gases were acetylene and oxygen.

### Data availability

The data that support the findings of this study are available from the authors on reasonable request, see author contributions for specific data sets.

### Porosity analysis from pycnometry and tomography

#### Helium Pycnometry

**Figure 3:**
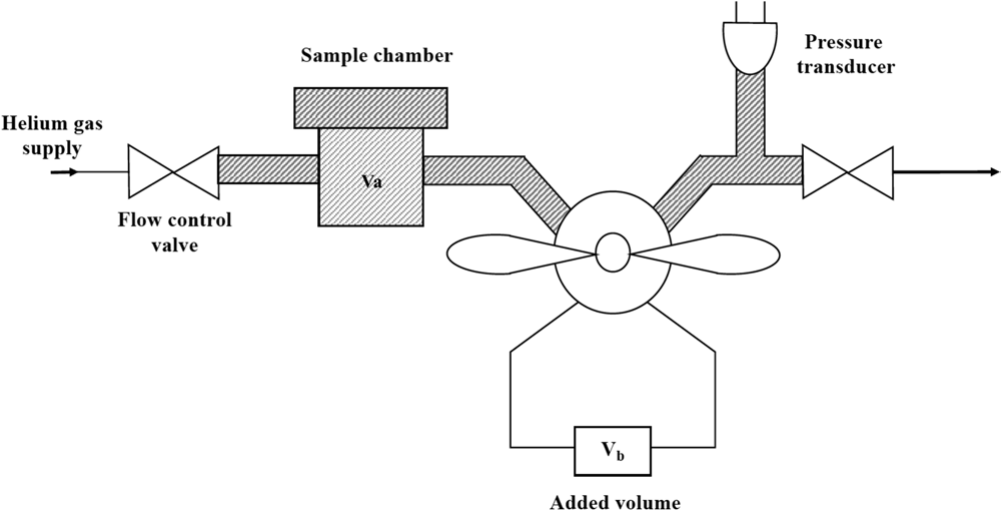
Schematic of the helium pycnometer^25^.

The volumes of samples were calculated using helium pycnometry (Ultrapyc 1200e, Quantachrome Instruments^16^) shown schematically in Fig. 3.

The method consists of placing a dry sample of known bulk volume, *V*_bulk_, in a sample chamber of known volume, *V*_a_, which is connected to an evacuated chamber of known volume, *V*_b_. Helium is introduced into *V*_a_ and the pressure, *P*_1_, set to an arbitrary value typically around 19 psi. The helium is released into *V*_b_ and allowed to equilibrate throughout both chambers, decreasing to a new stable level (*P*_2_). Using the ideal gas law, the volume of the sample, *V*_s_, can be calculated from Eq. 2^15^

2 $${V}_{{\rm{s}}}={V}_{{\rm{a}}}+{V}_{{\rm{b}}}(\frac{{P}_{2}}{{P}_{2}-{P}_{1}})$$

**Figure 4:**
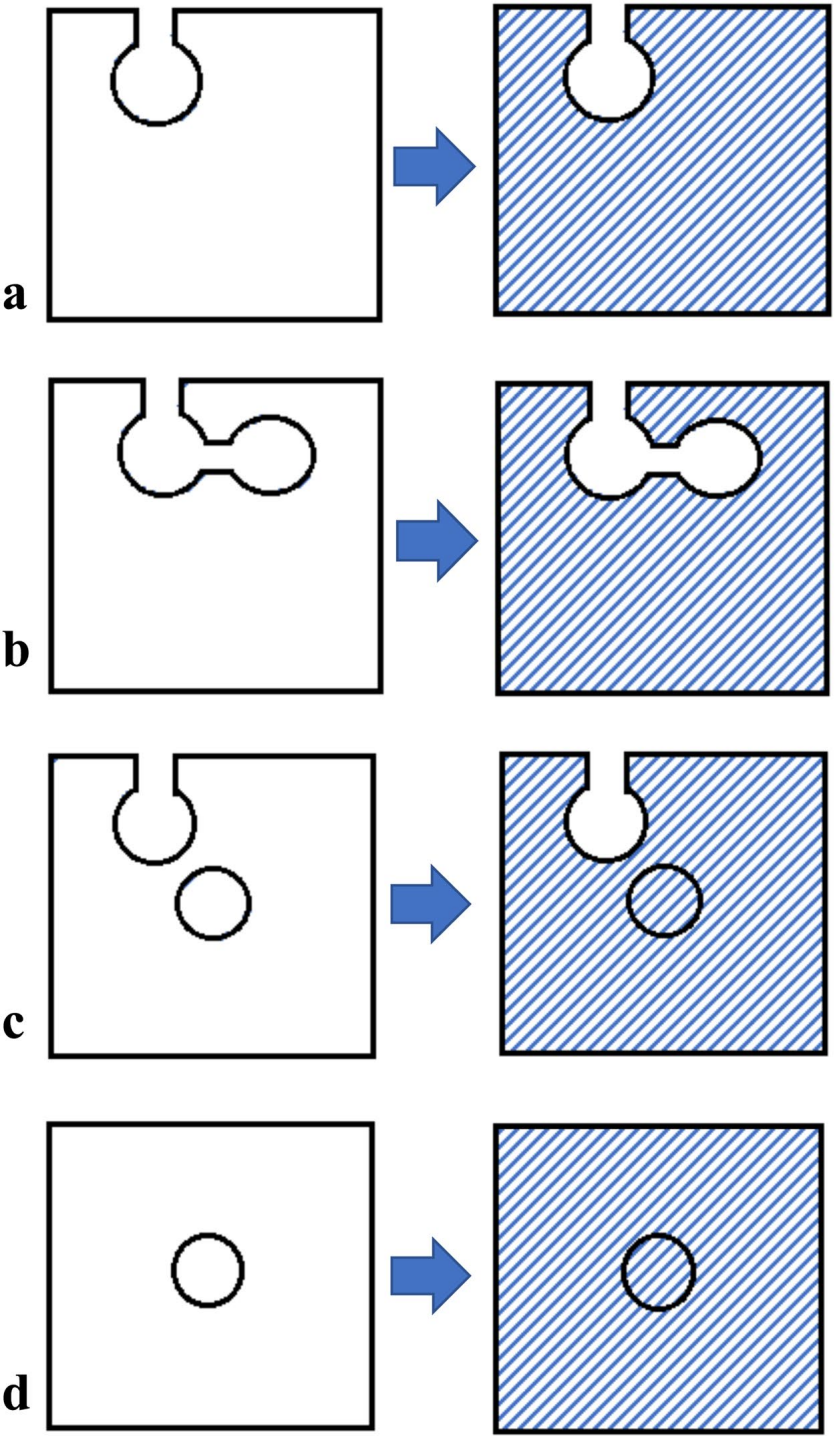
Schematics of samples with shaded areas indicating the volume measured by a helium pycnometer for pore types, (**a**) an open pore; (**b**) connected pores; (**c**) an open pore and an isolated closed pore; and (**d**) an isolated closed pore.

The density of the sample is determined from the sample weight and sample volume, *V*_s_. The pores inside the samples that are inaccessible to the helium are included in the *V*_s_, as schematically shown in Fig. 4.

The volume of the pores that are accessible to helium gas (open pores) can be calculated from

3 $${V}_{{\rm{o}}}={V}_{{\rm{bulk}}}-{V}_{{\rm{s}}}$$

The open porosity, *P*_o_, based on the bulk volume is computed as

4 $${P}_{{\rm{o}}}=\frac{{V}_{{\rm{o}}}}{{V}_{{\rm{bulk}}}}$$

The bulk volume, *V*_bulk_, of the sample is much greater than the coating volume, therefore the *P*_o_ is a small fraction. The open pore volume can also be calculated as a fraction of the film volume, *V*_f_. It should be noted that the copper substrate will also contain pores because of the manufacturing process^26^. Therefore, the open pore volume of the substrate, *V*_os_, should be deducted. The open film porosity, *P*_of_ based on the film volume is computed as

5 $${P}_{{\rm{of}}}=\frac{{V}_{{\rm{o}}}-{V}_{{\rm{os}}}}{{V}_{{\rm{f}}}}$$

To calculate the closed porosity, the mass of the deposited film, *m*_f_, is converted into film density, *ρ*_film_, by dividing the measured film thickness, *t*_f_ (volume based on thickness and area). *m*_f_ can be calculated by measuring the coated and uncoated samples. For the measurement of *t*_f_, computed micro tomography (CMT) is employed.

#### Computed Micro Tomography (CMT)

**Figure 5:**
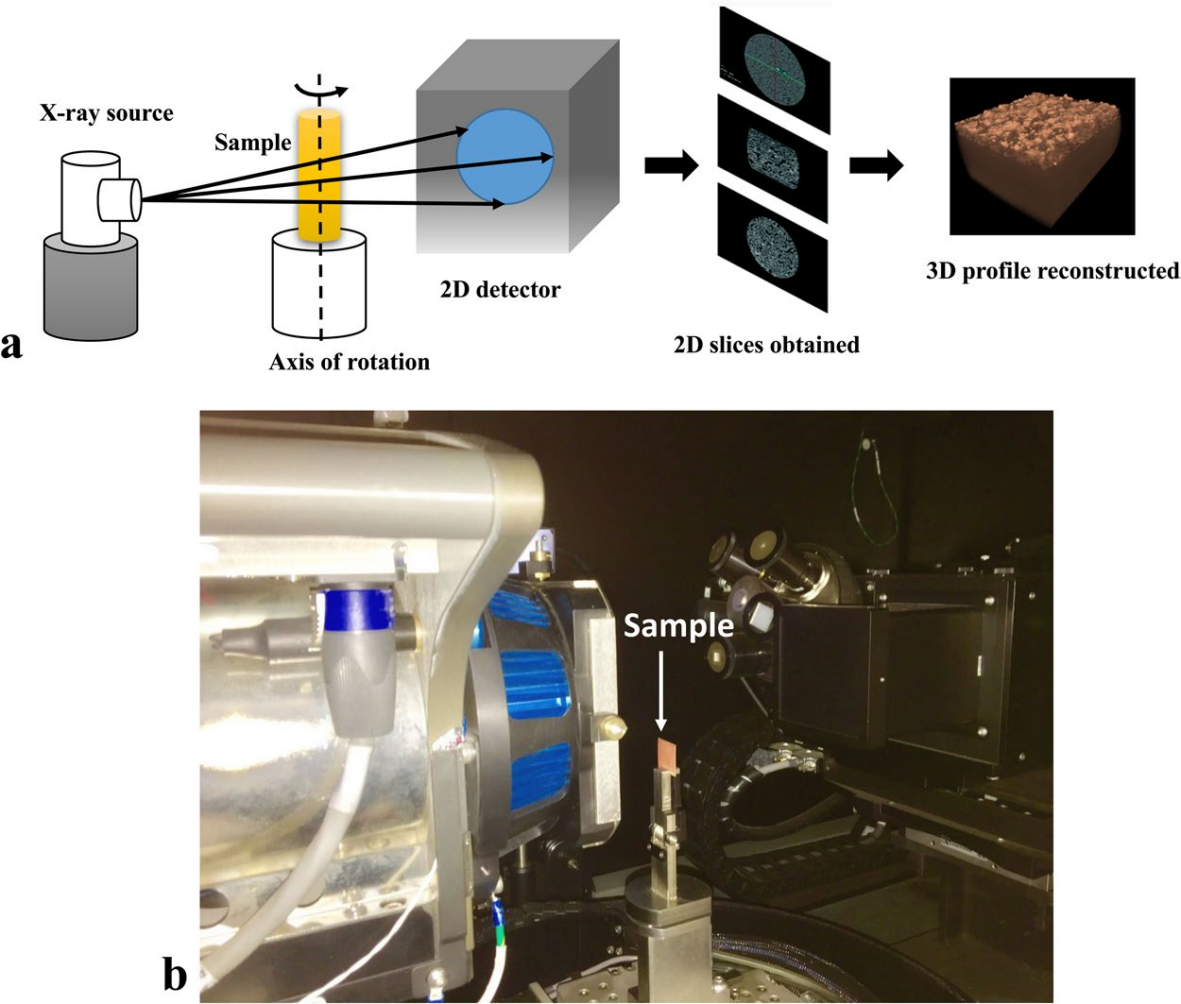
(**a**) schematic of computed micro-tomography; and (**b**) a coated sample mounted for micro-tomography measurements in an Versa XRM-500.

Thin film thickness of coated samples was measured by CMT (VersaXRM-500, Xradia, Zeiss, Jena, Germany). The coated sample mounted in the instrument for micro-tomography is shown in Fig. 5. The X-ray source energy was 140 kV, and a 4x objective with 3 s exposure time and HE3 filter for single image acquisition with 25–35% transmission. A total of 1601 2D transmission images was captured with sample rotation of 360°. The pixel size and the objective are set at 2 µm/pixel and 4X magnification, respectively. The collected 2D images were reconstructed into a 3D tomogram in XMReconstructor software (Xradia, Zeiss, Jena, Germany) using standard beam hardening correction with 0.7 size. From the 3D tomogram, the thickness of the coating is extracted from slices taken at various locations. Thickness measurement by CMT has the advantage over SEM or other imaging techniques as the former provides the film thickness at various locations without fracturing the sample and changing the morphology of pores.

From the measured thickness and the mass deposition, the film density is calculated from:

6 $${\rho }_{film}=\frac{{m}_{f}}{{t}_{f}}$$

To calculate the film porosity (total porosity), the density of the uncoated sample was evaluated as bulk density, *ρ*_bulk_. Consequently, the film porosity is calculated as

7 $${P}_{film}=1-\,\frac{{\rho }_{film}}{{\rho }_{bulk}}$$

Finally, the closed porosity is evaluated by taking the difference between film porosity and open porosity:

8 $${P}_{c}={P}_{film}-{P}_{{\rm{o}}}$$

### Telescope-goniometer

**Figure 6:**
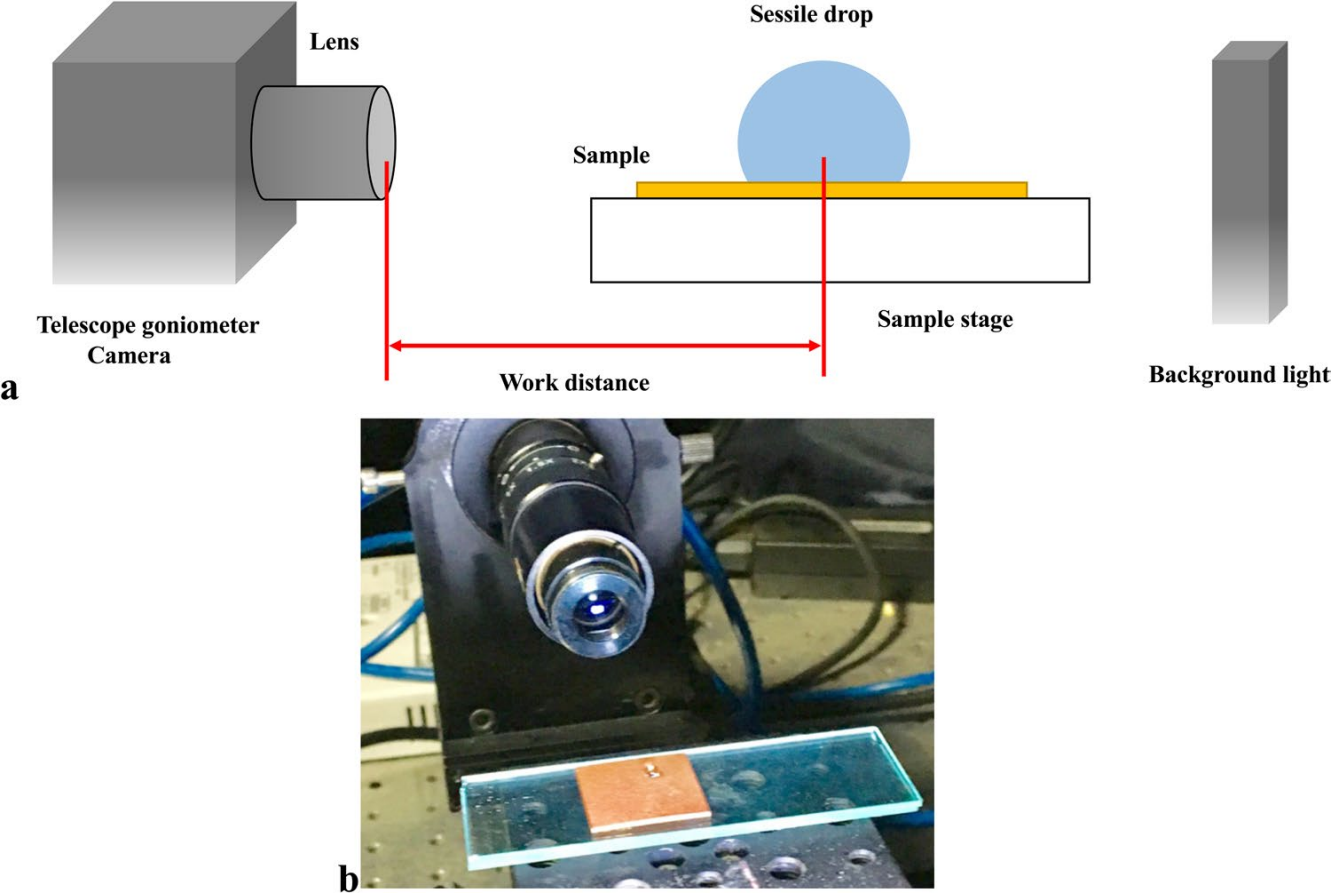
(**a**) a schematic of telescope-goniometer; and (**b**) photograph showing a liquid sessile drop formed on a coated surface.

**Figure 7:**
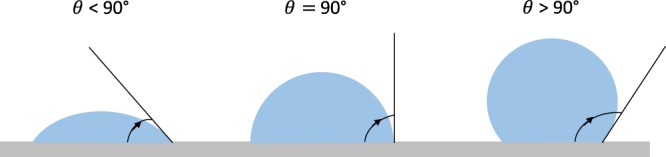
Illustration of contact angles measurement.

Contact angles are measured on three varieties of samples namely W1, P1, and UC. The telescope-goniometer consists of a flat stage to mount the sample, a micrometer pipette to form a water drop, an illumination source, and a telescope equipped with a protractor eyepiece. A schematic of telescope-goniometer is shown in Fig. 6a and a photograph showing a liquid sessile drop formed on a coated surface is shown in Fig. 6b. The measurement was achieved by merely aligning the tangent of the water drop profile at the contact point with the surface and reading the protractor through the eyepiece. The volume of water used for contact angle measurement was ~8 µL for each sample and it is assumed small enough to regard the coated film as flat. An integrated camera is used to take photographs of the drop profile so as to measure the contact angle. The measurement of contact angles formed on the solid surfaces is depicted in Fig. 7.

The surface roughness, *r*, is the ratio of the effective surface area due to roughness and the projected surface area. The Wenzel relation states that *r* is equal to the ratio of cosines of the contact angles of a liquid droplet on the rough surface (${\theta }^{\ast })$ and the contact angle of the same liquid on and ideal, flat surface $(\theta )$^27^:

9 $$\cos \,{\theta }^{\ast }=rcos\theta $$

## Results and Discussion

### Structural features of the thermal spray coated thin films

**Figure 8:**
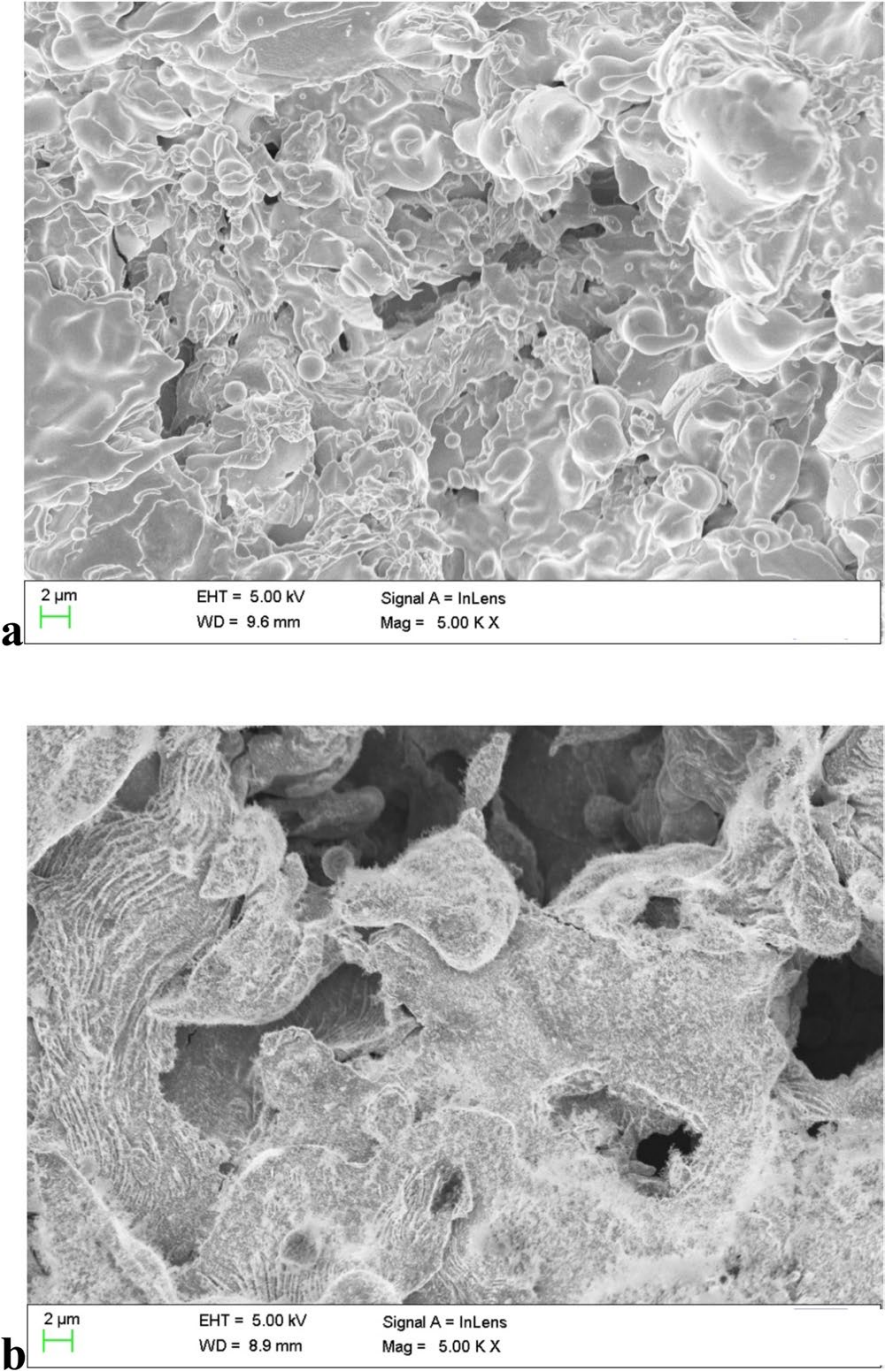
SEM images of (**a**) wire flame coating and (**b**) plasma coating.

To demonstrate the approach to define porosity and surface roughness, the surface features of the porous coatings were investigated. SEM images of wire flame and plasma coatings are shown in Fig. 8. It can be seen from Fig. 8a that the film deposited by the wire flame method has many pores with non-uniform shapes with diameters ranging from 1 μm to 10 μm. The pore diameters were measured using a calibrated scale bar on a SEM image. SEM images with scale bars corresponding to pore diameters are given as supplementary information. The pore diameters obtained from SEM images of wire flame and plasma coating ranges from mesopores (2–50 nm) to macropores (>50 nm)^13^.

Figure 8b reveals that the larger pores with irregular shapes are the plasma spray method. It also can be noticed from the SEM images that the film coating produced plasma method appears rougher than the wire flame method.

**Figure 9:**
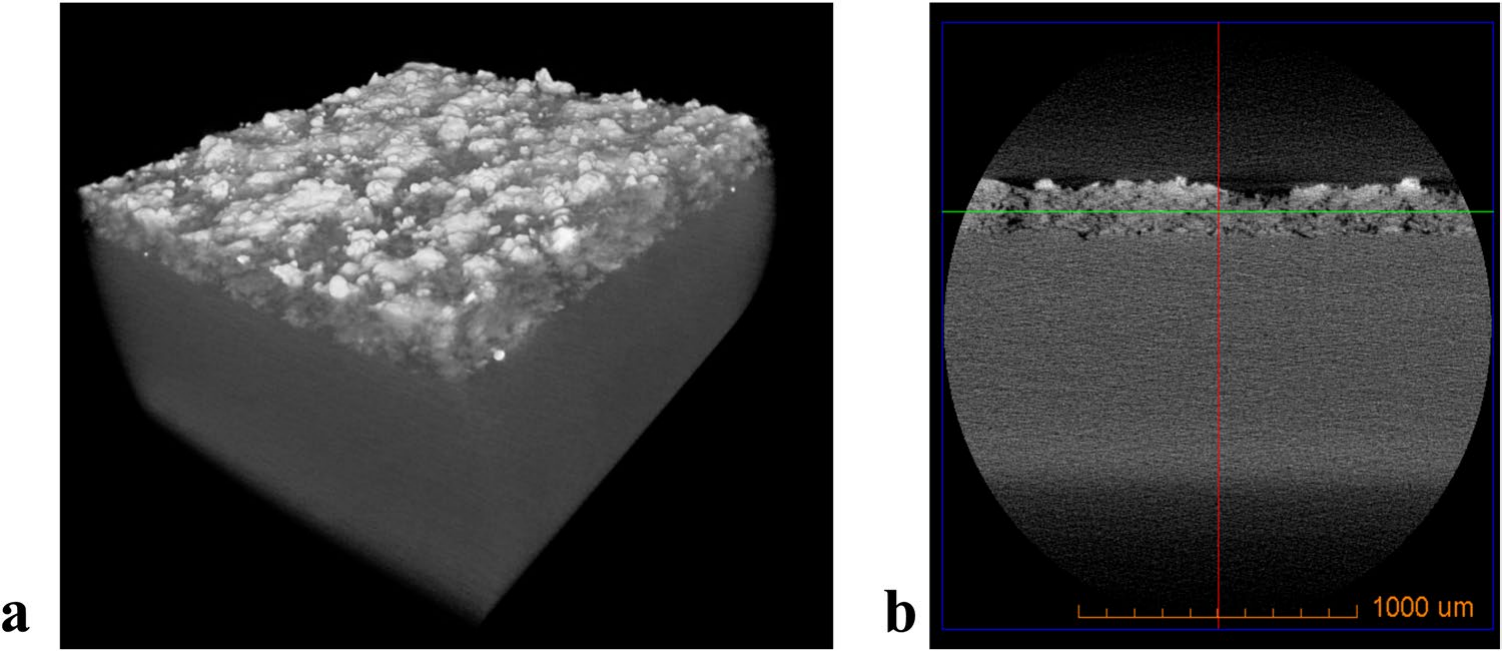
Images from computed micro tomography (CMT) showing (**a**) 3D tomogram and (**b**) thickness of the coating obtained from Xradia TXM3D Viewer.

**Figure 10:**
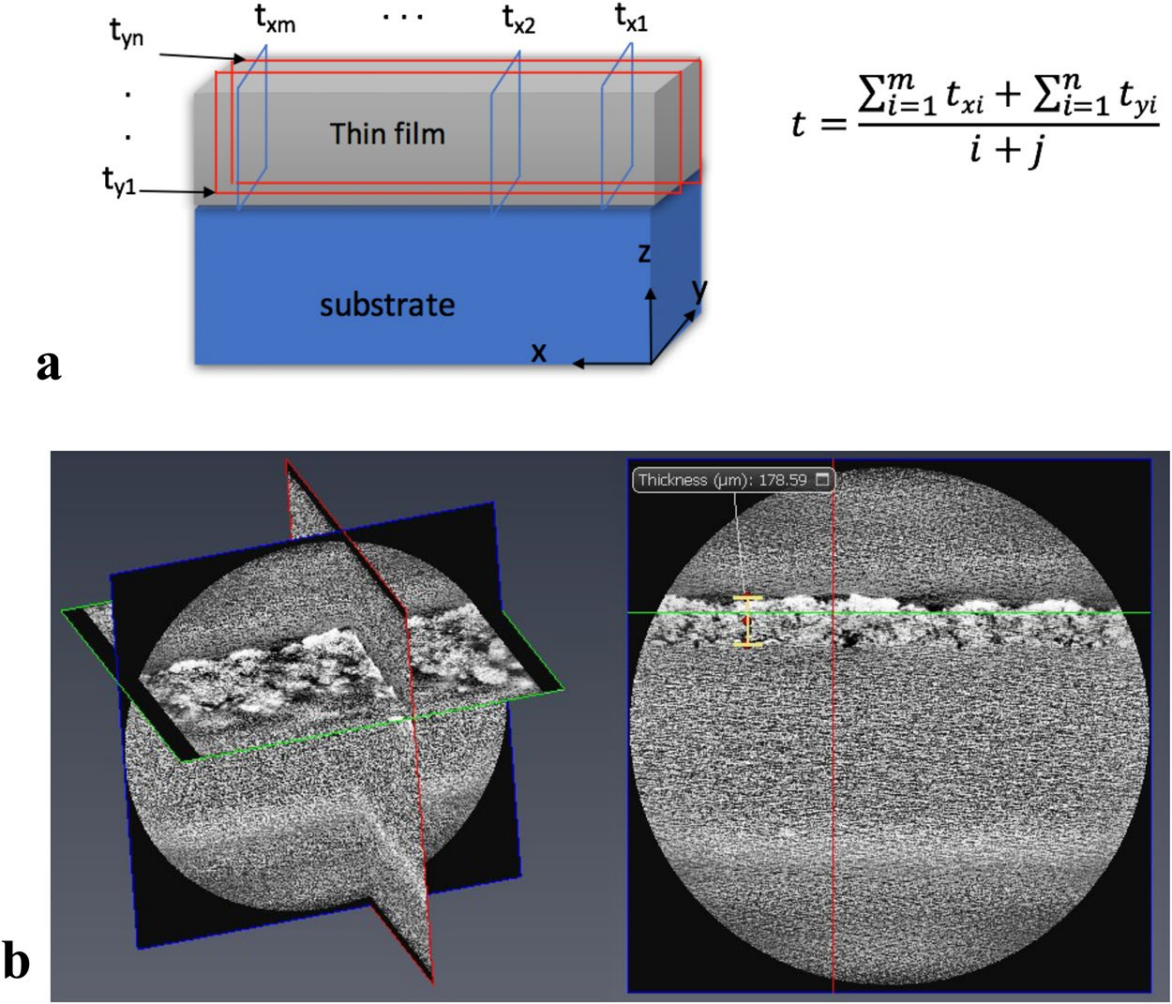
(**a**) A schematic showing the procedure to obtain the average thickness, and (**b**) orthogonal slices generated from image processing software AVIZO 9.4.0.

Figure 9 shows the images from computed microtomography (CMT). The 3D X-ray tomogram pictured in Fig. 10a shows the 3D image of porous thin film applied on a copper substrate, which is constructed from CMT. The sliced film of the coating in Fig. 9b obtained from TXM3D Viewer indicates that the pores are present beneath the surface as well. It also suggests that the pores are not homogenous throughout the depth of the film. The thickness (*t*) is calculated from taking the average of m slices in x direction and n slices in y direction as depicted in Fig. 10a.

### Film thickness

The thickness measurement was confirmed with another image processing software package (AVIZO 9.4.0) which read the TXM file of 3D tomogram generated from Xradia. The orthogonal slices are created from AVIZO as shown in Fig. 10b. Table 1 shows the measured thickness and standard deviation (average of x and y direction) for all coated samples.

**Table 1:** Details of thickness measure from CMT.

	Sample name			
	W1	W2	P1	P2
m,n number of slices in x and y direction	m, n = 4	m, n = 4	m, n = 4	m, n = 4
t, film thickness (μm)	200	230	180	201
σ, standard deviation	5.6	6.3	5.9	4.9

### Mass deposition, film density and porosity

**Figure 11:**
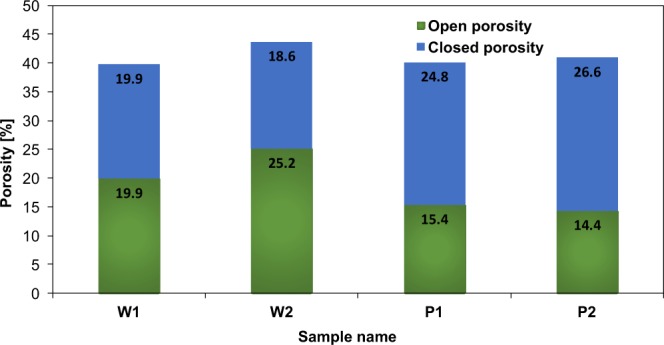
Open porosity and closed porosity for W1, W2, P1 and P2.

The mass deposited on the samples was calculated from the difference in the weight between the weight of each specific substrate measured before and after the coating was applied. Based on the mass of the deposited film and the measured thickness by CMT, the densities of the thin film samples were calculated using the Eq. 6. To calculate the film porosity (total porosity), the density of the uncoated sample was evaluated as bulk density, *ρ*_bulk_. Consequently, the film porosity is obtained from(Eq. 7) is:

Table 2 shows the measured data for all coated samples and Fig. 11 shows open porosity, closed porosity as well as film porosity (total porosity). For the sample W1, which is coated with wire flame thermal spray method, the mass of the film deposited is 0.3971 g, and the measured thickness is 200 μm (standard deviation =5.6), the density of the film amounts to 5.04 g/cc. The film porosity is calculated using Eq. 7 and taking percentage value, which is 39.77% ± 0.005%. Consequently, the open porosity and closed porosity amounts to 19.89% and 19.88% respectively.

**Table 2:** Measured parameters of all coated samples.

	Sample name			
	W1	W2	P1	P2
Substrate volume (cc)	0.295	0.295	0.295	0.295
Mass deposition (g)	0.3917	0.4301	0.3553	0.4071
Film thickness (μm) from CMT	200	230	180	201
Open pore volume of the substrate, *V*_os_ (cc)	0.0072	0.0072	0.0072	0.0072
Sample volume, *V*_s_ (cc)	0.3516	0.3563	0.3485	0.3563
Open pore volume, *V*_o_ (cc)	0.0229	0.0300	0.0181	0.0221
Film density, ${{\rho }}_{{film}}$(g/cc)	5.036	4.742	5.007	4.917

**Figure 12:**
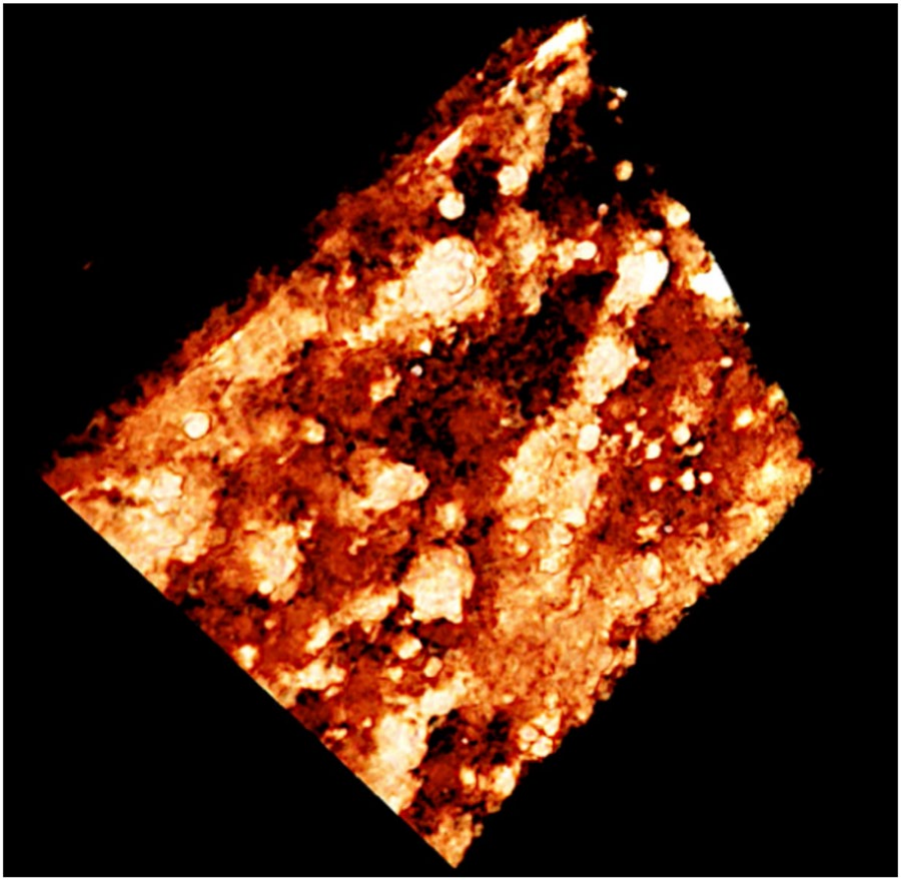
An image from computed micro tomography (CMT) showing 3D profile of the porous film.

For the sample W2, there is 8.3% more film deposited which corresponds to 15% increase in the thickness of the coating. Therefore, it resulted in 10% increase in the film porosity. The wire flame and plasma thermal spray methods produce highly porous thin films of porosity between 39–43%. This is also visually established in Fig. 12, which shows a 3D tomographic image of the film. A high resolution video file in MPEG format is provided as supplementary information.

### Surface roughness

**Figure 13:**
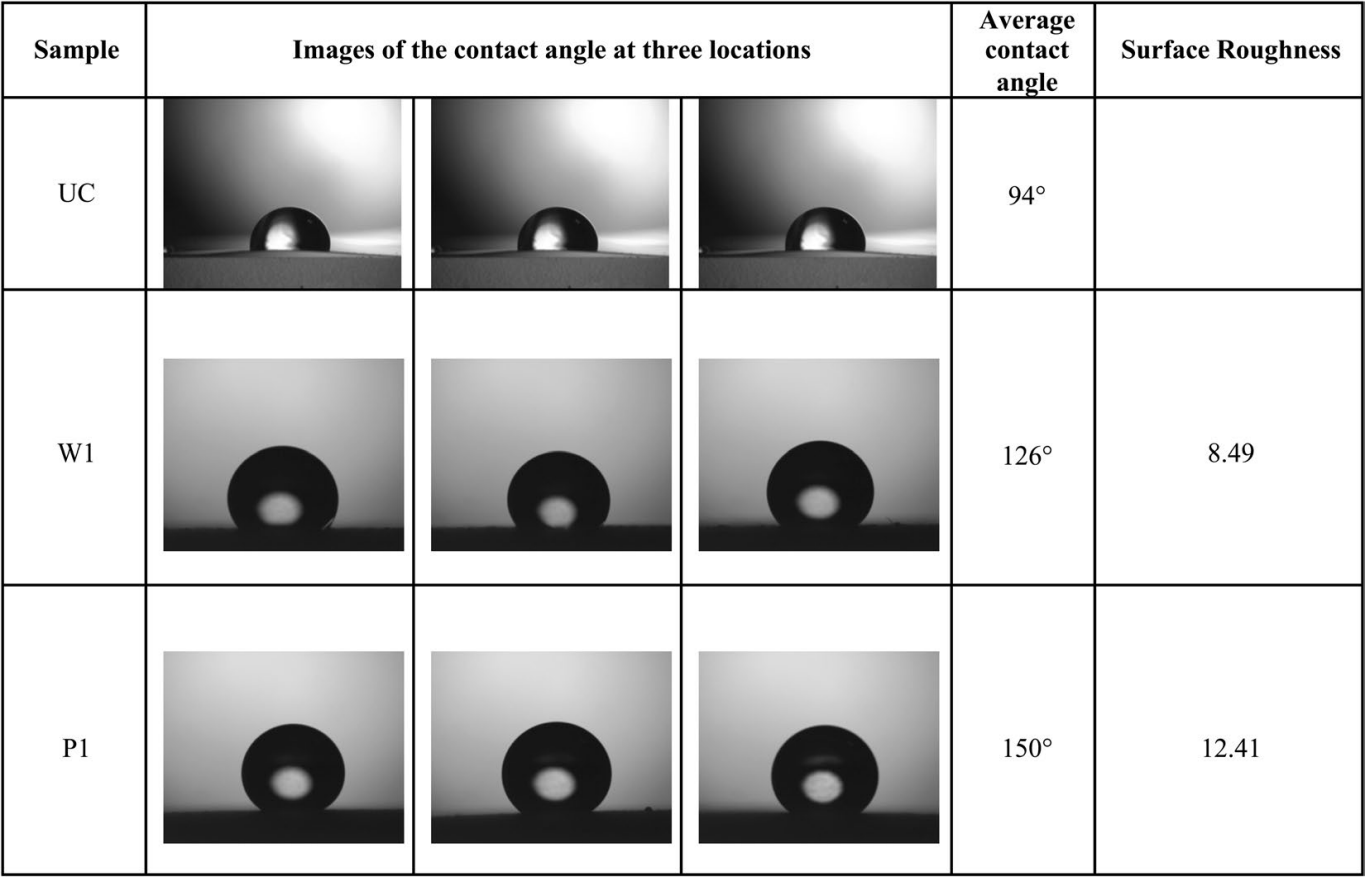
Wetting at three random locations on the porous copper coated surface.

In Fig. 13 the contact angles on an uncoated and coated copper substrate have been measured with a telescope-goniometer with precision of ± 2°. The average contact angle of water droplets on the uncoated copper was 94°, while the average contact angle on the wire flame coated surface and the plasma coated surface were 126° and 150°, respectively. The contact angles increased due the applied thin film. Therefore, the surface roughness enhances the hydrophobicity of the uncoated surface^23,28^. For the case of plasma coating, the surface has become super-hydrophobic^23^. By Wenzel’s relation, the wetted surface area of the porous copper coated thin film surface is 8.5 times and 12.4 times the wetted surface area of the uncoated surface for wire flame and plasma spray methods, respectively.

## Conclusion

The combination of pycnometry and computed micro-tomography was successfully demonstrated as a new tool to determine the porosity of thin films produced by thermal spray coating. Wenzel model is used to define the surface roughness ratio of thin films. Two types of thermal spray methods and four different thin film samples were evaluated for porosity and surface roughness ratio. The approach to determine the porosity relies on measuring the open porosity from helium pycnometer and film thickness from computed micro-tomography. The methodology to define the surface roughness ratio relies on measurement of the liquid droplet contact angle on the coated surface from a telescope-goniometer. The test results showed that the wire flame and plasma thermal spray methods produced highly porous thin films with porosities between 39–43%. The Wenzel method showed that the thermal spray coating increased the hydrophobicity of the thin film surfaces. For the case of plasma coating, the surface has become super-hydrophobic. This approach establishes that the porosity of thin films can be non-invasively determined and may also be applied to a wide variety of coated surfaces. In the future work, the porosity and roughness ratio of thin films will be assessed using only the data from CMT scanning.

## Electronic supplementary material

Supplementary Information

Video 1

Video 2
