# Supplementary material for: A New Approach to Compute the Porosity and Surface Roughness of Porous Coated Capillary-Assisted Low Pressure Evaporators
Source: Sci Rep. 2018 Aug 3;8:11708. doi: 10.1038/s41598-018-30090-9 (PMC6076235; doi:10.1038/s41598-018-30090-9)
Supplement: Supplementary file 1 — Supplementary Information [file 41598_2018_30090_MOESM1_ESM.pdf]

A New Approach to Compute the Porosity and Surface Roughness of Porous Coated Capillary-Assisted Low Pressure Evaporators

Poovanna Cheppudira Thimmaiah[1], Asish Kumar Panda[2], Upendra Kumar Pandey[3], Claire McCague[1], Pradip Dutta[3*] and Majid Bahrami[1][1]

[1]*Laboratory for Alternative Energy Conversion* (*LAEC*), School of Mechatronic Systems Engineering, Simon Fraser University, BC, Canada V3T 0A3

[2]*Materials Research Centre (MRC),* Indian Institute of Science (IISc), Bangalore, India 560012

[3]*Interdisciplinary Centre for Energy Research (ICER),* Indian Institute of Science (IISc), Bangalore, India 560012

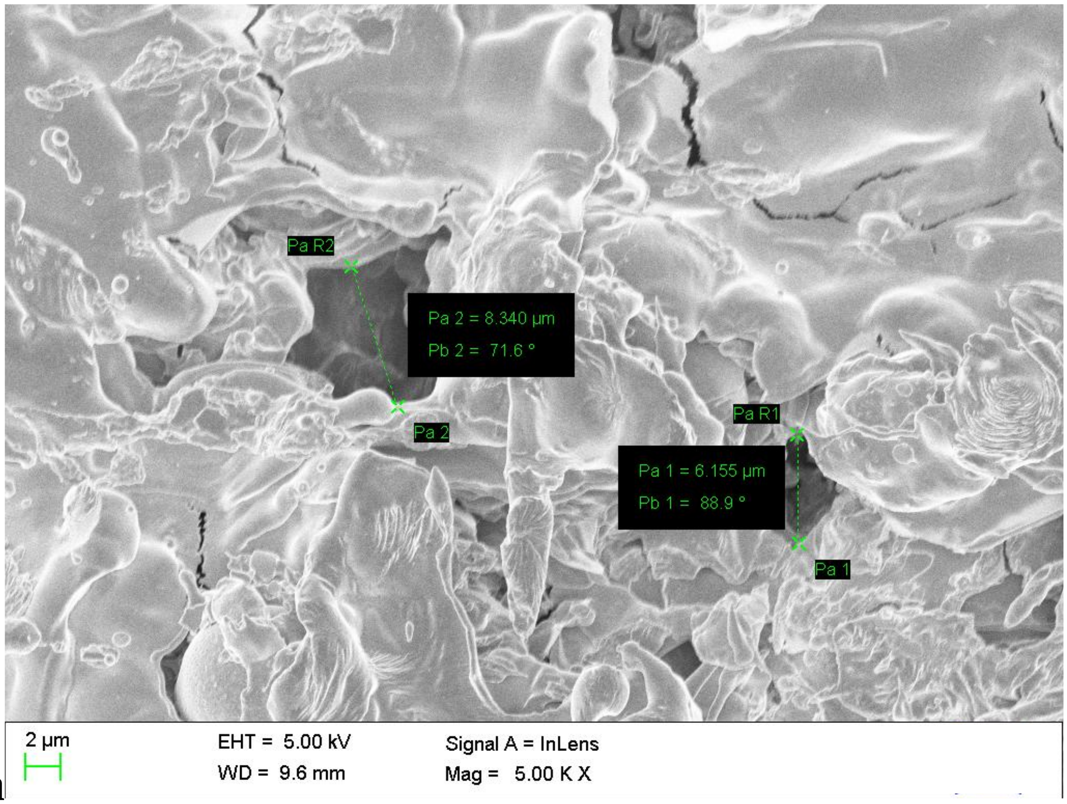

a

[1] Corresponding author
Tel.: +1 (778) 782-8538; Fax: +1 (778) 782-7514.
E-mail addresses pthimmai@sfu.ca (P. Cheppudira Thimmaiah), pradip@mecheng.iisc.ernet.in (P. Dutta), mbahrami@sfu.ca (M. Bahrami).

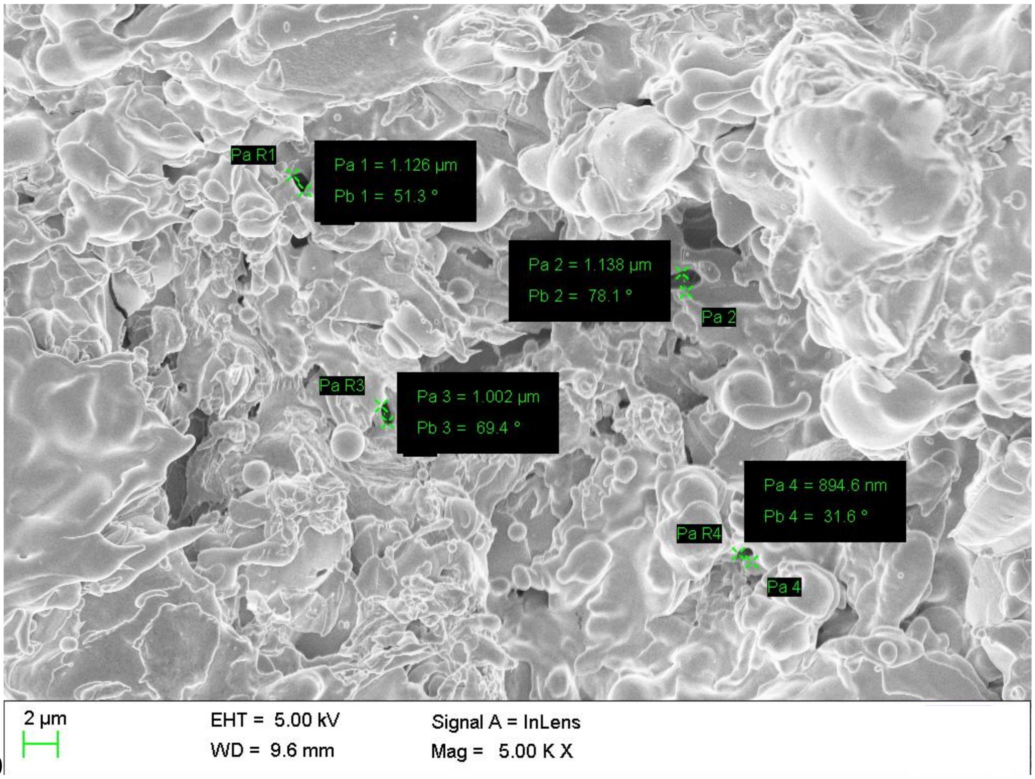

b

SEM images of wire flame coating at locations (a) and (b)

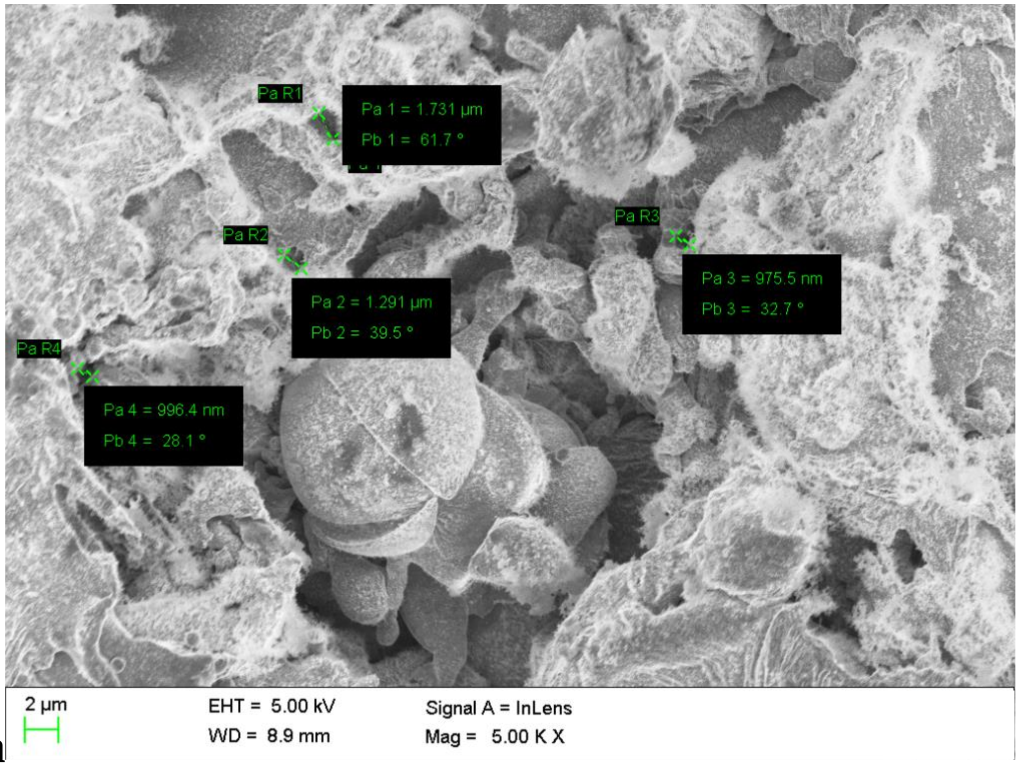

a

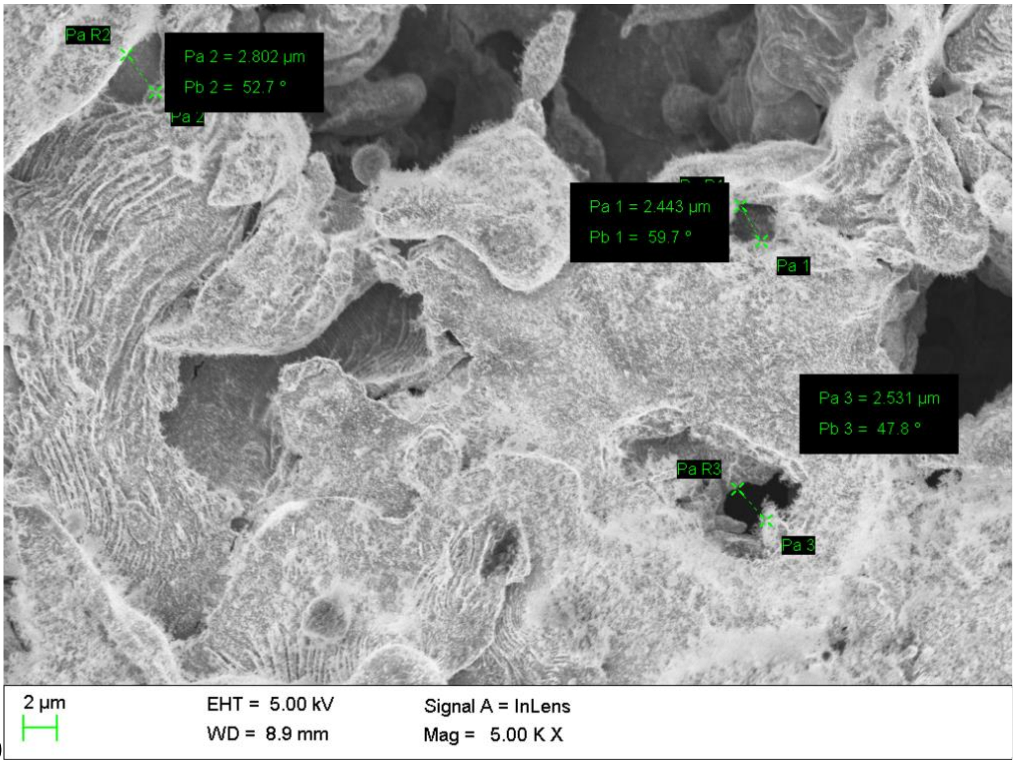

b

SEM images of plasma spray coating at locations (a) and (b)
